# A Case of Tick Bite Induced Babesiosis With Lyme Disease

**DOI:** 10.7759/cureus.17401

**Published:** 2021-08-23

**Authors:** Siddharth Bhesania, Kunwardeep Singh Arora, Michal Tokarski, Madiha Ariff, Fastina Khan, Janki Bhesania, Salman A Haq

**Affiliations:** 1 Internal Medicine, NewYork-Presbyterian Brooklyn Methodist Hospital, Brooklyn, USA; 2 Internal Medicine, Boston University School of Medicine, Boston, USA; 3 Internal Medicine, Dow University of Health Sciences, Karachi, PAK

**Keywords:** babesia micoti, lyme disease, ixodes tick, co-infection, pancytopenia

## Abstract

The Ixodes tick may transmit multiple pathogens, Lyme disease being the most common. Early detection of tick bites and using prophylaxis measures is the key to prevent tick bite-associated diseases like babesiosis, anaplasmosis, and Lyme disease. It is recommended to follow preventive measures like using diethyltoluamide (DEET) on the skin, applying permethrin on clothes while visiting the tick-infested areas. Co-infection is an uncommon occurrence but still representative in endemic areas. If there is delayed initiation of therapy in these kinds of patients, there may be dire consequences that may require aggressive therapy. Clinicians should consider co-infection when suspecting tick-borne disease which can prove to be fatal if not addressed promptly. Here, we present the* *case of a 72-year-old female with atypical symptoms, who was found to have coinfection with Lyme disease and Babesiosis on serology testing and peripheral smear and* *was diagnosed and treated promptly.

## Introduction

Lyme disease (LD) is the most frequent infection transmitted by the Ixodes tick; however, other diseases such as babesiosis and anaplasmosis are also possible. Ticks have been attributed to illness for more than a century. The earliest recognition of illness caused by Ixodes ticks came in the early twentieth century when a Swedish dermatologist observed that the bite of an *Ixodes ricinus* tick was linked with erythema chronicum migrans, a distinctive skin lesion surrounding insect bites [1]. Babesiosis was first reported in humans in 1957 [2]. Since then, the number of newly identified diseases and health risks connected with Ixodes ticks has grown substantially. The incidence of dually infected Ixodes ticks appears to be higher in ticks from North American and European locations where Lyme disease is prevalent, with a reported prevalence of ≤28% [3]. Humans coinfected with LD and babesiosis appear to have more intense, prolonged symptoms than those with LD alone [3]. We present a case where a patient with atypical symptoms was found to have co-infection with Lyme disease and Babesiosis. Co-infection should be suspected if the patient presents with the atypical clinical picture of a single pathogen or lack of improvement with therapy after 48 hours for the presence of co-infection.

## Case presentation

**Figure 1:**
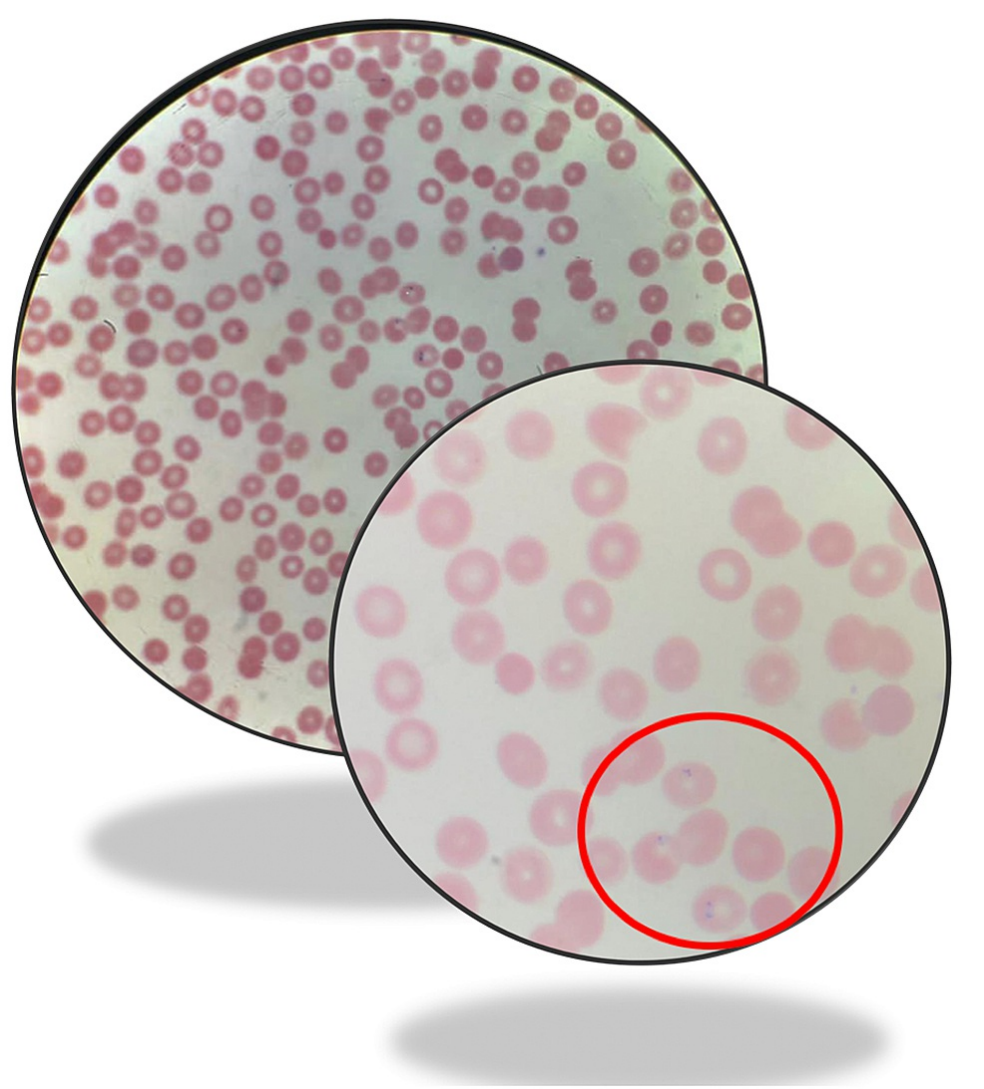
Peripheral blood smear showing Babesia microti in our patient's blood sample.

A 72-year-old female presented with complaints of three weeks of persistent fever [maximum temperature (T-Max): 104 F], chills, nausea, and productive cough of yellow sputum. The patient traveled to Connecticut six weeks before the presentation, stayed there for 20 days. She denied any known tick bite or rash but during the previous year, she had two tick bites. She denied any other acute or chronic symptoms or any obvious sick contacts. She also denied any known immune deficiency or malignancy. She worked in a tailoring company. On initial presentation, the patient was afebrile and hemodynamically stable. Physical exam was unremarkable, with no rash, no lymphadenopathy, or evident tick bite. Initial blood work showed pancytopenia. Chest X-ray (CXR) and urinalysis (UA) were not suggestive of infection. The manual peripheral blood smear showed *Babesia microti* in RBCs (Figure 1).

The patient was started on azithromycin, atovaquone for babesiosis, and doxycycline to treat Lyme disease with initial suspicion of co-infection and a plan to deescalate once the Lyme disease was ruled out. Due to atypical presentation, Lyme serology and peripheral thick smear were evaluated for parasite confirmation. Lyme serology showed positive immunoglobulin M (IgM) antibodies. The patient was continued on all the antibacterial medication. Initial blood smear showed 1.7% RBCs infected with *Babesia microti *which trended down to 0.4% and <0.1% infected RBCs on repeat blood smear on days 4 and 7. The patient was discharged home to complete the course for 14 days. 

## Discussion

**Figure 2:**
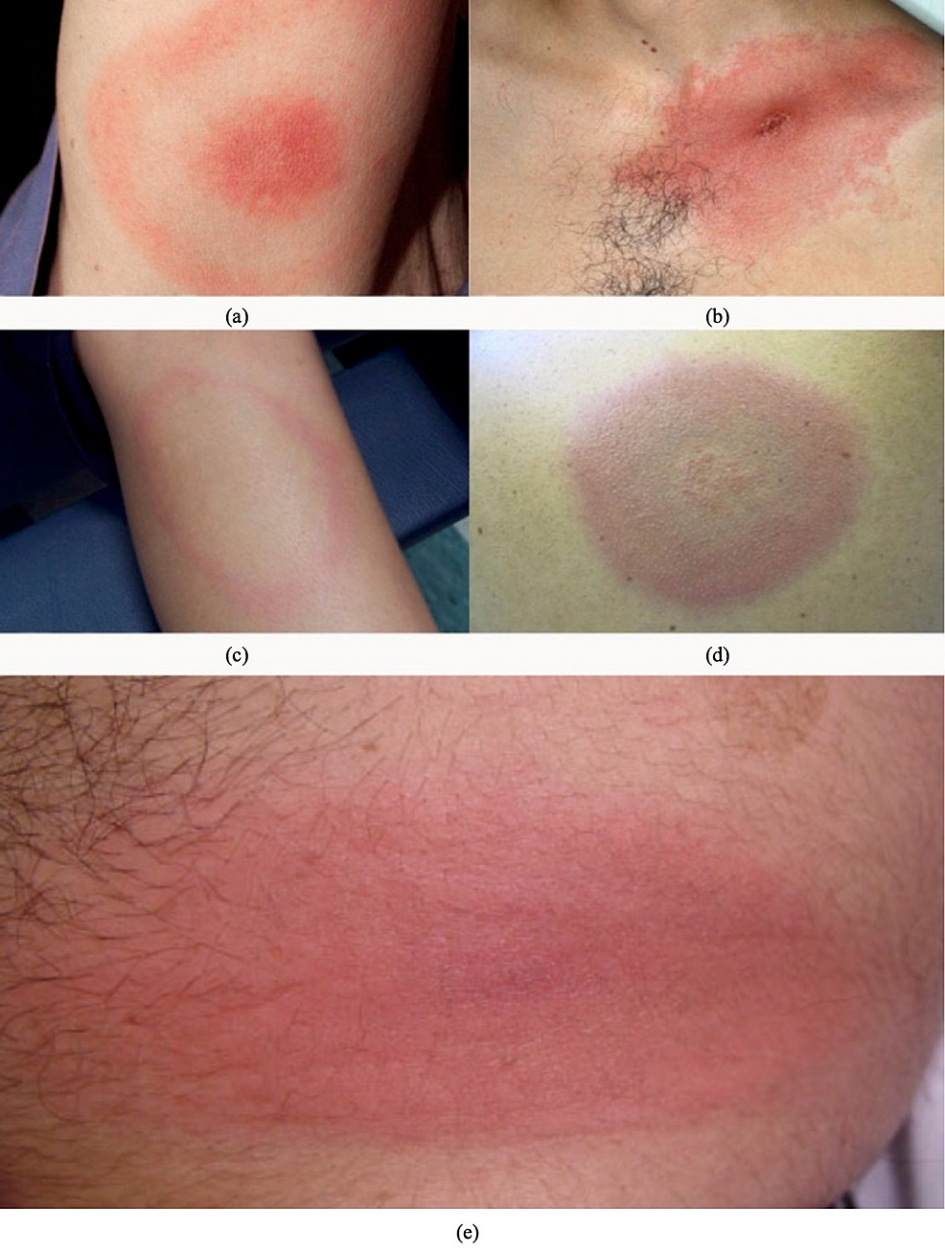
Various types of rashes that can occur in Lyme disease. Content source for images (a) to (e): Reference [9] (a) - Classic Lyme disease rash (circular expanding rash with target-like appearance). (b) - Expanding rash with central crust (expanding lesion with central crust on chest). (c) - Expanding rash with central clearing (circular, expanding rash with central clearing). (d) - Bluish hued rash, no central clearing (Bluish hued without central clearing). (e) - Red, oval plaque (Red, expanding oval-shaped plaque on the trunk).

Ixodidae have been discovered as human spotted fever vectors [4,5]. Spotted fever symptoms typically appear 4 to 10 days after a tick bite and vary depending on the species involved [6]. Typical symptoms include fever, headache, malaise, muscle pain, rash, and local lymphadenopathy [7]. A distinct skin lesion at the location of the tick bite, known as eschar, may develop in the majority of cases with spotted fever [7] (Figure 2). Lyme disease can affect multiple systems of the body and produce a series of symptoms, including erythema migrans, fever, arthritis, neurologic and heart damage, and may continue to develop severe chronic consequences such as neurological symptoms and disability [8].

**Figure 3:**
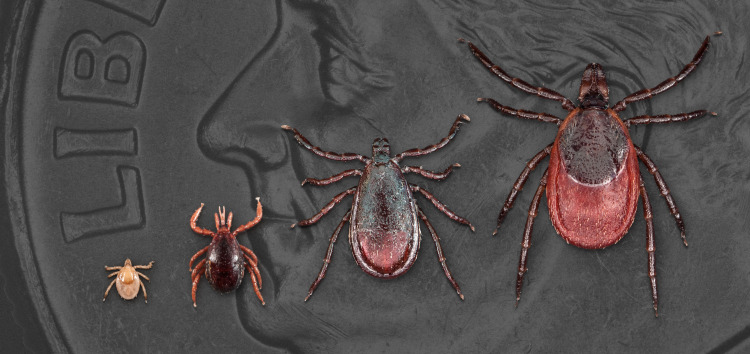
All life stages of Ixodes scapularis Image source: Centers for Disease Control and Prevention [10] The dime shown in the background is for scale.

The black-legged tick (*Ixodes scapularis*) (Figure 3) is common in the northeastern and upper midwestern United States. They are responsible for the transmission of diseases including Lyme, anaplasmosis, babesiosis, and Powassan. The seasons with the greatest danger of tick bites are spring, summer, and fall. 

**Figure 4:**
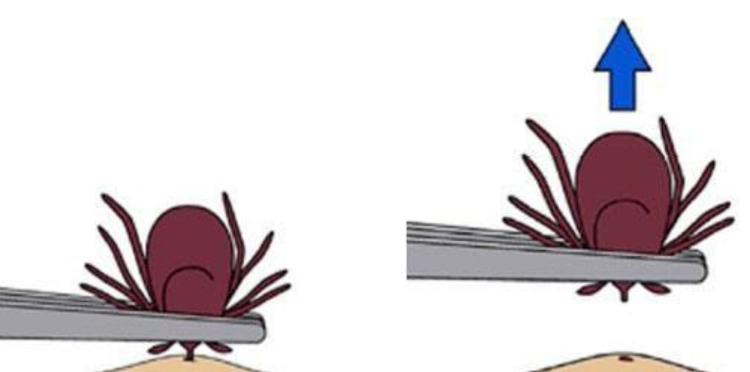
Method of tick removal from the surface of the skin Image source: Centers for Disease Control and Prevention, National Center for Emerging and Zoonotic Infectious Diseases (NCEZID), Division of Vector-Borne Diseases (DVBD) 2019 [11].

When a patient comes with an adhered tick on their body, tick removal is suggested. An alcohol pretreatment is used to clean the location where the tick has adhered. It is recommended to grasp the head rather than the body, as removing the body will leave the head attached (Figure 4).

If the head is not removed, it becomes a breeding ground for infection, and removal becomes a much more difficult task [12]. It is recommended to pull out the tick from the skin abruptly and directly while expecting the tick to take a little area of skin with it. Following removal, the skin should be cleaned routinely and a topical antibiotic, such as bacitracin, should be used, followed by application of a wound dressing to the affected region. Prophylaxis is recommended when all the following circumstances exist: (1) the attached tick is an adult or nymphal *Ixodes scapularis* tick, and is attached for more than 36 hours, (2) prophylaxis started within 72 hours of the time that the tick was removed, (3) the local rate of infection of these ticks with *Borrelia burgdorferi* is greater than 20%, and (4) doxycycline is not contraindicated. Prevention of tick bites to avoid complications include: avoiding tick-infested areas, using diethyltoluamide (DEET) on the skin according to label directions, applying permethrin to clothing, wearing clothing in a downward cascade [13]. 

In a case report, a 67-year-old woman had a fever, rash, and myalgias for 5 days, which persisted after a 21-day course of amoxicillin for Lyme disease (*Borrelia burgdorferi* infection). She was also found to have pancytopenia. Further workup for pancytopenia showed *Babesia microti* as a provocative factor for her symptoms. The patient improved after appropriate babesia treatment [14].

Our case has similarities with this case in that the patient had a persistent fever for three weeks along with pancytopenia. The unique feature of our case was the atypical presentation with no rash and no joint pain, but the patient had only constitutional symptoms like weakness and occasional fever, which later confirmed with positive IgM Lyme disease serology. Our patient improved with disease-specific antibacterial treatment.

## Conclusions

Lyme disease is the most frequently diagnosed tick-borne disease. Patients from endemic areas may benefit from being screened for a co-infection. The clinical picture of tick-borne disease is extremely variable. Patients with delayed initiation of therapy may require aggressive therapy. Cases with severe hemolytic anemia, disseminated intravascular coagulation, respiratory failure, renal failure erythrocyte apheresis should be considered. Clinicians should consider co-infection when suspecting tick-borne disease which can lead to fatal consequences if not addressed promptly.
